# Digital mental health tools for use by individuals in opioid use recovery: An academic scoping and commercial review

**DOI:** 10.1371/journal.pdig.0001544

**Published:** 2026-07-30

**Authors:** Jessica N. D’Arcey, Saleena A. Zedan, Leah Tackaberry-Giddens, Cheyenne McIntyre, Sana Junaid, Toni-Rose Asuncion, Sean A. Kidd

**Affiliations:** 1 Schizophrenia Division, Centre for Addiction and Mental Health, Toronto, Canada; 2 Department of Psychology, University of Toronto Scarborough, Scarborough, Canada; 3 Department of Psychology, Ball State University, Muncie, Indiana, United States of America; 4 Department of Psychology, Concordia University, Montreal, Canada; 5 Department of Psychiatry, University of Toronto, Toronto, Canada; The University of Hong Kong, HONG KONG

## Abstract

Opioid use disorder (OUD) is a global public health crisis that has steadily worsened despite significant efforts. Maladaptive opioid use can have significant negative impacts, yet a minority receive evidence-based treatments. Leveraging common personal technologies like smartphones and computers may help extend access to such services. The current study aims to describe the landscape of personal technology use in opioid use recovery by combining an academic scoping review with a review of commercially available smartphone apps and devices. The scoping review provides an overview of research examining feasibility and efficacy, whereas the commercial review investigates whether available technology is supported by peer-reviewed research or regulatory approval. Four databases were searched for the scoping review (i.e., PubMed, PsychINFO, EMBASE, and MEDLINE) alongside Apple and Google Play stores for the commercial review. Two independent reviewers evaluated the studies. The academic search yielded 937 articles, of which 34 were included, and the commercial review found 21 apps and devices. The included studies show good acceptance and feasibility; however, research reporting on efficacy outcomes is mixed and nascent. Of the commercially available interventions, only eight underwent peer-reviewed evaluation, and only three had received healthcare body approval at the time of the search. Thus, more research is needed to ascertain the efficacy of such interventions, especially in underrepresented groups such as women, minority groups, and insecurely housed people. Additionally, given the limited data identified and the legal and medical risks associated with OUD, further consideration of safety and privacy is warranted. Further, since commercialization has vastly outpaced peer-reviewed research, we suggest amendments to regulatory procedures that consider the risks associated with the intended population, rather than just the intended use. Lastly, we offer suggestions to guide clinical decision-making regarding the use of digital tools in their practice.

## Author summary

The present study is a scoping and commercial review describing the existing landscape of digital health tools that leverage commonly owned personal technologies, like smartphones, computers and tablets, to support people recovering from opioid use. We identified 34 studies, most of which were uncontrolled pilot studies, highlighting good acceptance and feasibility in this population. Included randomized trials and other experimental designs show mixed efficacy findings. Primary targets for interventions were abstinence and adherence-based. The commercial review identified 21 available interventions, with only 8 reporting supporting research. Our review highlights important gaps and directions for future research, regulatory evaluations, and clinical decision-making regarding the integration of technology into opioid addiction recovery. A significant finding is that the use of health apps in this field is in its infancy, with limited research confirming their effectiveness, safety, and privacy. This finding, paired with the finding that the vast majority of commercially available apps (i.e., those clients can already download) were not evidence-based or had regulatory approval, led us to recommend regulatory amendments and clinical caution. Key considerations that affect the appropriateness of such technological interventions include potential legal consequences related to data security, adequate safety resourcing, clarifying medical-legal responsibility, treatment target fit, and navigation of acute risk scenarios.

## 1. Introduction

Substance use disorders (SUD) have increased by 33.5% between 1990 and 2017, with global drug use disorder reaching 7.7 million [1]. More specifically, during this period, Opioid Use Disorder (OUD) rose from 47.18% to 53.1%, which was the fastest-growing SUD globally. A pressing issue underscored by the rising rates of lethal overdose [2]. Fatal opioid overdoses are rapidly increasing across several countries, including the USA, the United Kingdom (UK), Canada, Australia, and across Europe [2]. For example, in 2022, deaths due to overdose were over 100,000 in the United States (US) alone [3]. For those living with OUD, the personal burden is immense, impacting several important domains such as mental and physical health and well-being, relationship functioning, employment, and residential status [4]. Further, problematic opioid use is often comorbid with other serious mental health conditions, including depression, schizophrenia, and bipolar disorder [5]. In addition to impacts on individuals, there are significant economic consequences associated with OUD. The overall economic cost of OUD in the US in 2017 was estimated to be $1.02 trillion, with the majority of costs resulting from reduced quality of life (i.e., lost productivity, incarceration) and the value of life lost due to fatal overdoses, which accounted for more than half this amount [6].

This global public health crisis has steadily worsened despite significant efforts in public health efforts and advancements in treatments [7]. Public health efforts have included investments in law enforcement [8,9], public education [10], and efforts to create safer supply and drug-use contexts [11]. Regarding treatment, there is strong evidence that supports medications for opioid use disorder (MOUD) such as Opioid Agonist Treatment (OAT) or Opioid Maintenance Treatment (OMT) as the ‘gold standard’ treatment for OUD. A review of clinical practice guidelines reported the most common recommendation for MOUD was for OATs, such as buprenorphine, followed by methadone, with varied recommendations for psychosocial interventions [12].

MOUD is associated with reduced the risk of OUD-related death, including overdose, suicide, HIV, hepatitis B and C infections, and injuries [13,14]. Despite researchers attesting to the benefits of sufficiently dosed and uninterrupted MOUD treatment [13], access to MOUD remains low, with studies from 2019 reporting approximately 30% [15] and 20% in 2021 [16] of individuals with OUD accessing MOUD in the US. There are several barriers to MOUD, including variable access and coverage of MOUD, as well as stigma and financial costs associated with attending appointments [17].

Additionally, there are growing calls for the integration of evidence-based psychotherapy interventions alongside MOUD to improve retention and treatment of comorbid mental health conditions [18]. An estimated 60% of individuals with OUD have co-occurring mental illness, 36% with mild-moderate, and 24% with serious mental illness (SMI), yet 47% of individuals with mild-moderate and 21% of individuals with SMI do not receive psychosocial or behavioural interventions [19]. A recent review of psychosocial interventions suggested that cognitive behavioural therapy (CBT) and Educational Behavioural Counselling may be most efficacious compared to treatment-as-usual (TAU) for opioid use, whereas educational behavioural counselling may be most impactful on treatment discontinuation [20].

Other, more flexible and cost-effective means of supporting retention may be digitally-supported care, which has been shown in other areas of substance use [21,22] and mental health conditions [23,24] to improve adherence to treatment and engagement in services. Arguably, the most accessible digital support strategies are those that leverage personal technologies (i.e., commonly owned devices like smartphones, computers, tablets, etc.). However, like other fields, questions remain regarding the pace of commercialization and whether the evidence supports the widespread use of these tools.

The commercialization of digital mental health tools leveraging personal technologies has increased rapidly. In the UK alone, there are 21,000 health apps and 3,857 mental health apps on Apple and Google Play stores [25]. However, most of these commercially available digital supports for mental health have little to no supporting evidence. Research examining digitally supported care (i.e., the use of digital mental health tools within existing care structures) for substance use has also climbed rapidly in the past decade, with one review identifying over 3000 abstracts from 2015 to 2022, with the mobile phones identified as the most popular technology [26]. Other reviews examining digital supports for substance use identify between 17 and 22 eligible studies, with the minority being randomized control trials (RCTs) [27,28], except for one study, which identified twelve RCTs [29]. Several reviews examining the clinical impact of these interventions have found promising results regarding reduced substance use [27,28,30] and improved treatment engagement [27]. However, these reviews also state that evidence regarding effectiveness is limited, especially for drugs other than alcohol (e.g., cannabis, stimulants, and opioids) [29,31].

More specifically, digital tools for opioid use are often missed in reviews examining substance use broadly or categorized with other drug types, making it difficult to assess the available research on interventions specifically targeting opioid use. For instance, many reviews have not identified any RCTs examining opioids specifically [28,29,31], one identified one [27], and other studies either only included mixed drug groups which included opioids [27] or combined their results with studies examining other drug types [26]. One meta-analysis of internet interventions for substance use specifically did look at opioid use separately and showed overall positive effects on opioid abstinence at post-treatment visits (n = 606, g = 0.36, CI = 95%) [32]. Further, to these authors’ knowledge, there has only been one systematic review (*n* = 20 RCTs) examining opioids, which found that approximately 50% of included studies showed positive effects on abstinence and 20% on treatment retention [33]. However, given the narrow (i.e., on a particular technology, study design) or broad focus (i.e., general substance use that often overlooks opioid use specifically) of existing reviews, it is difficult to understand the overall state or landscape of existing literature examining the use of personal technologies in opioid use recovery.

### 1.1 The current study

The current scoping review aims to provide an overview of research investigating the feasibility and effectiveness of digitally supported interventions leveraging personal technologies for OUD. To the best of our knowledge, such a review has yet to be undertaken, and capturing the field’s current state will be important for providing future research directions and practice guidance. Broadly, this review provides an overview of existing technologies designed to support recovery in OUD and identifies gaps and limitations within the existing evidence for their use. More specifically, we examine the feasibility and safety of digitally supported interventions, to aid in the understanding of their potential role within existing care structures. We provide an overview of existing evidence on effectiveness and future directions for research in this area. Lastly, we focus on the commercially available technologies to better understand the evidence for their use, including funding, research setting (i.e., academic vs. industry), and study design.

## 2. Methods

Given the novelty of the field and the limited number of RCTs examining each technology type, the current study combines a scoping review paired with a commercial review to provide the most complete picture of existing research and commercially available digital supports leveraging personal technologies. A scoping review “aims to systematically identify and map the breadth of evidence available on a particular topic” regardless of study methodology or data type [34], which is best suited for a description of the research landscape as proposed here. The scoping review followed updated scoping review methods [35–37], which build on Arskey and O’Malley’s (2005) foundational work. Additionally, the PRISMA Scoping Review Checklist was used to guide the structure, methodology, and results [38]. The protocol for this scoping and commercial review is available on the Open Science Framework at DOI 10.17605/OSF.IO/PHRBJ.

### 2.1 Methods for the academic scoping review

#### Inclusion and exclusion criteria.

Studies were included if they examined digitally delivered or supported interventions for opioid/substance use involving personal technologies (i.e., commonly owned personal devices, including smartphones, computers, tablets, smartwatches, etc.). Interventions, including TAU, delivered synchronously over the phone or via video conferencing (i.e., telemedicine) were not included in this review as these interventions are not the same as digitally supported interventions. In this review, digitally supported interventions are defined as novel interventions with a technological component in addition to or external to clinician-delivered care. Virtual reality was outside the scope of this study as it typically requires technological devices that are not commonly owned. The population of interest includes individuals with OUD; as such, if studies used a mixed substance use sample, at least 50% of the sample must have included individuals with OUD. Specific criteria are included in Table 1. Studies were excluded if the intervention target was not opioid or substance use, the intervention was not supported by technology, the sample was less than 50% OUD/opioid misuse, or the outcome was not an outcome of interest. There were no exclusions for the study design; studies with either a priori quantitative and qualitative hypotheses examining the feasibility or effectiveness of a digitally supported intervention were included in the current review. Some additional exclusions include publications not published in English, publications that do not present novel empirical research (e.g., editorials, reviews, commentary), and publications with no full text available (e.g., conference presentations). No full-text articles were identified in the search that were inaccessible. Search terms included publication years between 2010 and 2023.

**Table 1:** Study inclusion criteria.

Population Diagnosis	Digital Platform	Outcome Target	Study Methodology
• Opioid Use Disorder • Opioid Abuse • Opiates • Narcotics • Opioid related disorders • Opioid addiction • Medication Assisted Treatment	• Mobile Applications • Text Messaging • Web-Based • Video Conferencing • Wearables • Blended Interventions	• Opioid Use/Abstinence • Overdose Rates • Symptoms • Community and Social Functioning • Service Engagement • Illness-Management	• Randomized Controlled Trials • Pilot Trials • Qualitative Studies • Feasibility Studies • Protocol Papers

#### Search methods.

Cochrane, PROSPERO and OSF databases were searched for existing reviews on the topic, with no results. Four core databases were searched on January 2^nd^, 2023: PubMed, PsychINFO, EMBASE, and MEDLINE. Google Scholar (the first five pages) was checked as a grey literature source, and references of included studies were hand-searched to ensure an adequate breadth of search. The following is a summary of the search terms that were used:

Powered by OVID (PsycINFO, EMBASE, MEDLINE): exp Methadone Maintenance/ or exp “Opioid Use Disorder”/ or Opioid Use*.mp. AND (sms or short message* service* or texting or text message*).mp. OR (mobile apps* or smartphone app* or digital health) OR (eHealth or mHealth or mobile health or internet intervention or web-based treatment or web-based intervention or wearable*).mp.

Detailed search terms for each OVID-powered database are included in S1–S3 Figs in S1 Text in the Supporting Information.

Non-OVID Databases (PubMed): ((((opioid use disorder) OR (Opioid dependence)) OR (heroin dependence)) OR (opioid abuse)) AND (((((sms text messaging) OR (smartphone app*)) OR (website)) OR (internet based)) OR (wearable))

#### Review procedures.

Studies resulting from the initial search were exported and de-duplicated in EndNote [39] before being uploaded to an online blinded-reviewer platform called Covidence [40], where they were de-duplicated a second time. All studies were reviewed by at least two reviewers at both the title and abstract stages to determine initial inclusion and again at the full-text stage to confirm inclusion. Inter-rater agreement was investigated using proportionate agreement (i.e., the percent of agreement between two raters), and Cohen’s Kappa was calculated based on raters’ initial independent ratings. If there were any disagreements regarding inclusion among reviewers, the last author made the final determination.

#### Data extraction and analysis.

Two reviewers extracted data independently, and a third reviewer resolved any discrepancies. Overarching data, such as the number of studies published by year and technology type, will be displayed graphically. Data extracted includes information on the study or research report included in the review (i.e., author, year, research setting, funding, and country), key sample characteristics (i.e., diagnosis, % white ethnicity, age, clinical setting, relapse, and overdose rates, special considerations such as unhoused status), and intervention design (i.e., type and role of technology, workflow, target, methods, mode of delivery). The main outcomes include the feasibility and effectiveness of the included interventions. Data on feasibility include study attrition, intervention dropout, technology use, participant feedback/user ratings, and safety indices. Data extracted regarding effectiveness includes change scores (i.e., symptom changes, relapse rates, overdose rates, and medication adherence), effect sizes, and p-values. Data regarding safety indices and privacy were also extracted. Safety indices include a discussion of safety measures to mitigate harm as well as safety outcomes such as serious adverse events (SAEs) and adverse events (AEs). Similarly, any data pertaining to the privacy measures taken to mitigate the risk of breach (e.g., reviews of privacy measures, third-party privacy testing, encryption, etc.) or any privacy outcomes (e.g., privacy breaches or reported concerns) were extracted. However, the values reported will vary depending on the study design. Data analysis is primarily descriptive and followed PRISMA guidelines [36]. Using the research questions as a guide, a narrative synthesis of the literature was conducted to provide an overview of the state of research in the area. Results are organized under the following categories: Study Information, Technological Intervention Characteristics, Feasibility, and Effectiveness.

### 2.2 Methods for the commercial review

A search of available apps that targeted opioid use disorder was completed across several platforms using the keywords “Opioid Use Disorder,” “Digital Health,” and “OUD.” The platforms included a general Google search, the Apple iOS and Google Play stores, and the PsycINFO database. Commercialization information was extracted from the identified technologies, including commercialization dates, patent information, regulatory registrations, the countries where the technologies were available, and any relevant collaborators. Associated research not included in the academic review was cited and summarized in the commercial review section. Additionally, funding sources, data transparency, and conflict-of-interest statements were extracted for all studies included in both the academic and commercial reviews.

## 3. Results

### 3.1 Academic scoping review results

**Fig 1:**
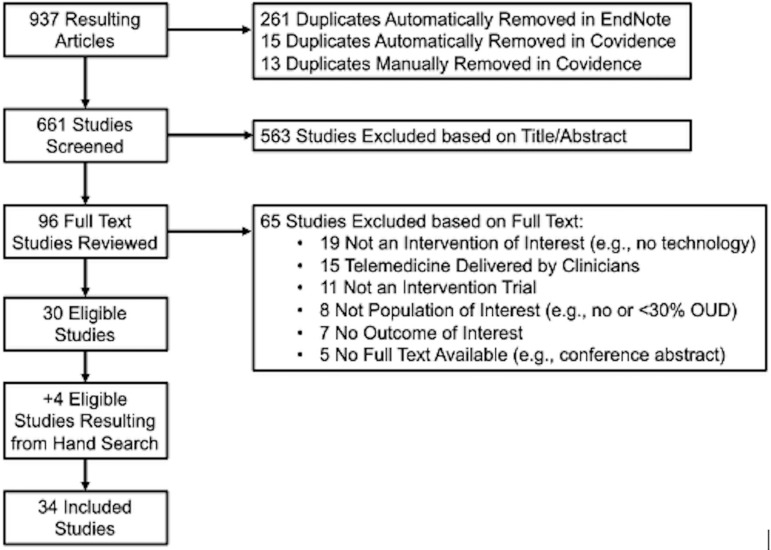
PRISMA breakdown of review and resulting articles.

Our initial search of the four core databases yielded 937 articles, of which 289 were identified as duplicates and removed using automated screening processes. A total of 661 articles underwent manual, blinded screening by at least two reviewers. After a review of the title and abstract, 563 were excluded, and after the full-text review, another 65 were excluded. For a detailed breakdown of the reasons for exclusion, please see Fig 1.

**Fig 2:**
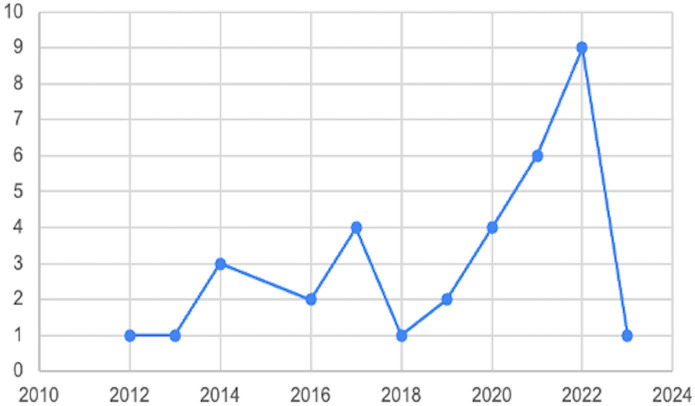
Publications over time.

**Fig 3:**
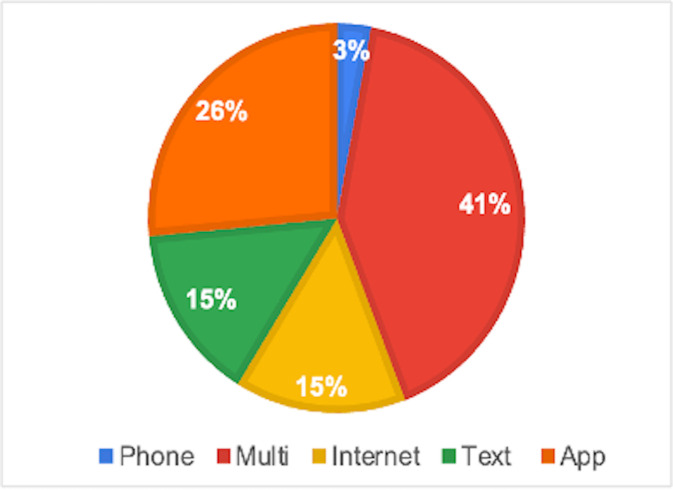
Technology types in included studies.

**Study information.** Inter-rater reliability was moderate (i.e., within a moderate range between.41 and.60) at both the title and abstract (87% proportionate agreement, 0.62 Cohen’s Kappa) and full-text phases (80% proportionate agreement, 0.44 Cohen’s Kappa), indicating reasonable to good agreement. Any conflicts (i.e., disagreements about inclusion) were resolved upon review by the team and PI. Challenges with ratings included that descriptions of interventions were often unclear, making it difficult to understand whether and what technology was involved and whether an intervention was being delivered (i.e., survey studies about a potential intervention, the use of technology in care, co-design studies, etc.). The included studies (*n* = 34) were published between 2012 and 2023, with most published between 2019 and 2022 (*n* = 21); see Fig 2. The vast majority of included studies were published in the USA (*n* = 31), with one published in Singapore, the UK, and Portugal. Most studies were conducted in populations with OUD who were being treated with OAT, such as methadone or buprenorphine. It is difficult to ascertain the exact number of clinical settings using OAT, depending on the description provided. Samples primarily consisted of individuals with a diagnosis of OUD, with a small minority targeting substance use broadly, with a subset of individuals with OUD (*n* = 2). Samples tended to be comorbid, with polysubstance use not being an exclusion criterion. Most studies were conducted with predominantly white (*n* = 22 studies with >50% white, n = 19 studies >70% white) male samples (*n* = 25 studies >50% male, *n* = 14 studies >70% male). Despite housing insecurity being reported in studies as a factor influencing the feasibility of interventions, 25 studies did not report on the living status of participants. Six studies reported that a relevant proportion of participants were experiencing homelessness, no fixed address, or unstable housing (range = 14–56%, *M* = 33%). Technologies used to deliver interventions included those leveraging multiple types of technologies (“multi-technology”; *n* = 14), smartphone applications (*n* = 9), internet-based (*n* = 5), SMS text messaging-based (*n* = 5), and telephone-based (*n* = 1); see Fig 3. Most studies, 28 (90.32%), reported that participants were additionally engaged in some form of substance use treatment during the study period (Table 2).

**Table 2:** Overview of included studies.

First Author	Country	Technology Type	Sample Size	% OUD	Clinical Setting	Study Design (Primary Aim)
Acosta, 2012 [41]	USA	Internet-Based	160	100	Initial Methadone Maintenance Treatment	RCT (Efficacy)
Bosse, 2022 [42]	USA	Smartphone App	31	100	Telehealth-based OUD Treatment	Qualitative Focus Group (Development)
Brooklyn, 2022 [43]	USA	Multi-Technology	58	100	Opioid Treatment Program	Pre-Post Non-Controlled (Feasibility)
Christensen, 2014 [44]	USA	Internet-Based	170	100	Local Clinics	RCT (Efficacy)
DeFulio, 2021 [45]	USA	Smartphone App	124	100	Outpatient Addiction Centre	Non-Randomized Retroactive (Efficacy)
DeFulio, 2023 [46]	USA	Smartphone App	20	100	Medication Assisted Treatment Centres	Pre-Post Non-Controlled (Feasibility)
Flickinger, 2022 [47]	USA	Smartphone App	25	100	MAT Clinics	Qualitative Pilot (Feasibility)
Gamito, 2017 [48]	Portugal	Smartphone App	14	NR	Therapeutic Community for Drug Dependence Treatment	RCT (Efficacy)
Godersky, 2020 [49]	USA	Multi-Tech	14	100	Outpatient Clinic	Pre-Post Non-Controlled (Feasibility)
Guarino, 2016 [50]	USA	Multi-Tech	50	100	Outpatient Methadone Maintenance Treatment Clinic	RCT (Feasibility)
Gustafson, 2016 [51]	USA	Multi-Tech	440	100	Addiction Treatment Centres	RCT Protocol
Hodges, 2022 [52]	USA	Multi-Tech	25	100	Office-Based Opioid Treatment	Pre-Post Non-Controlled (Feasibility)
Holtyn, 2021 [53]	USA	Multi-Tech	41	100	Community Agencies	RCT (Efficacy)
Kawasaki, 2022 [54]	USA	Multi-Technology	15	100	Opioid Treatment Program	Pre-Post Non-Controlled (Feasibility)
Langdon, 2020 [55]	USA	Multi-Technology	80	100	Outpatient Addiction Treatment Services	Development and RCT Protocol
Langdon, 2021 [56]	USA	Multi-Technology	24	100	Outpatient Addiction Treatment Services	Qualitative (Development)
Langdon, 2022 [57]	USA	SMS Text Message	8	100	Outpatient Clinic (Forensic)	Qualitative (Development)
Marsch, 2014 [58]	USA	Internet-Based	160	100	Outpatient Methadone Maintenance Treatment Clinic	RCT: Secondary Analysis (Feasibility)
Metrebian, 2021 [59]	United Kingdom (UK)	SMS Text Message	10	100	Opioid Agonist Treatment Providers	RCT (Feasibility)
Moore, B., 2013 [60]	USA	Telephone-Based	36	NR	Outpatient Methadone Maintenance Treatment Clinic	RCT (Feasibility)
Moore, B., 2017 [61]	USA	Multi-Technology	127	100	Opioid Treatment Program	RCT (Feasibility)
Moore, S., 2019 [62]	USA	Internet-Based	60	At least 50%	Community-Based Adolescent Substance Abuse Treatment Program	Qualitative (Development)
Proctor, 2022 [63]	USA	Smartphone App	10	100	Addiction Treatment Centre	Qualitative (Feasibility)
Radick, 2023 [64]	USA	Multi-Technology	78	100	Office-Based Opioid Treatment	RCT (Feasibility)
Scherzer, 2020 [65]	USA	Smartphone App	40	100	Outpatient Hospital-Affiliated Clinic	Pilot Trial Protocol
Schramm, 2020 [66]	USA	Multi-Technology	80	100	Office-Based Opioid Treatment	RCT Protocol
Schulman-Oliver, 2018 [67]	USA	Multi-Technology	12	100	Office-Based Opioid Treatment	Pre-Post Non-Controlled (Feasibility)
Suffoletto, 2017 [68]	USA	SMS Text Message	17	100	Emergency Department	Pre-Post Non-Controlled (Feasibility)
Tofighi, 2017 [69]	USA	SMS Text Message	93	100	Office-Based Opioid Treatment	Pre-Post Non-Controlled (Feasibility)
Tofighi, 2022 [70]	USA	SMS Text Message	50	100	Office-Based Opioid Treatment	Pre-Post Non-Controlled (Feasibility)
Tsui, 2021 [71]	USA	Multi-Technology	78	100	Office-Based Opioid Treatment	RCT (Efficacy)
Waselewski, 2021 [72]	USA	Smartphone App	25	100	MAT Centres	Pre-Post Non-Controlled (Feasibility)
Zhang, 2018 [73]	Singapore	Smartphone App	30	NR	Inpatient Detoxification Centre	Feasibility Protocol
Zhang, 2019 [74]	Singapore	Smartphone App	30	57	Inpatient Detoxification Centre	Pre-Post Non-Controlled (Feasibility)

*Note: SMS = short message service, Multi-Tech = interventions that leverage multiple types of technologies, App = smartphone application, MAT = medication assisted treatment, OUD = opioid use disorder, NR = not reported, RCT = randomized controlled trial.*

#### 3.1.1 Reported feasibility & real-word considerations.

***Feasibility of research*:** Data extracted on feasibility include study attrition, participant feedback/user ratings, safety, technology engagement, and privacy indices. Regarding study feasibility metrics, 25 (73%) of the 34 included studies reported study attrition. Overall, the approximate average reported attrition rate was 21%. Another important feasibility consideration was whether devices were provided to participants who did not own the necessary devices. Eight studies reported providing devices (including mobile phones, tablets, headphones, and electronic pill dispensers), with two studies reporting that most devices were returned [49,67]. The other studies did not report whether the devices were returned, damaged, stolen, or not returned for any other reason. All but one of the open pilot trials (*n* = 1/11; [46]) included positioned feasibility as their primary outcome. In addition to the aforementioned feasibility indicators, studies focused on the usability of the app, measured via user satisfaction and engagement with the technology, outlined below.

***User satisfaction & descriptive feedback*:** Thirteen studies (38%) reported user satisfaction ratings. Generally, most study participants reported that the digital interventions were highly useful and easy to use. More specifically, some studies found that participants were motivated to use technology to support their recovery [74] and that the use of technology would help them manage their recovery and symptoms [67,74]. Another study examined satisfaction ratings over time and reported comparable scores at four and 12-month time points [45]. Alternatively, Godersky et al. (2020) reported user concerns regarding technology use, including comfort with the technology, privacy while using it, and difficulties accessing the technology (e.g., logging in).

Regarding descriptive feedback, five studies provided positive qualitative data supporting the acceptability, ten studies provided qualitative data supporting the usability, and five studies provided qualitative data supporting the perceived helpfulness of the technological interventions. Seven studies reported popular features, including the use of positive reinforcement, appointment reminders/ability to reschedule, daily journaling/reflections/check-ins, goal tracking, and stories of lived experience [42,49,50,59,60,68,72]. Alternatively, seven studies reported qualitative feedback on technology improvements, such as enhanced community engagement, use of multimedia, increased resources, and personalized content [42,50,54,57,60,68,72]. Further, six studies identified barriers to use (e.g., access to cellphones, discomfort using video, technical issues, offline accessibility, privacy concerns, and non-accessible language) and clinical integration (e.g., asynchronous vs. synchronous communication and alerts to clinicians for urgent support) [42,49,50,54,57,68,69,72].

***Technology engagement*:** Measures for technology engagement, including frequency of use, were highly variable in the included studies. A total of 26 studies reported metrics of overall technology engagement. Measures of engagement included the frequency of technology use (*n* = 18), the number of days users interacted with the intervention (*n* = 4), and the number of completed modules/responses (*n* = 4). Engagement metrics and outcomes also tended to differ by technology type. For text message interventions, the response rates were variable. Tofighi et al. (2022) reported that 88% of users responded to at least one message, whereas other studies reported lower rates, ranging from 0% to 12% across the study period [59]. Further, two studies reported attenuated responses to text messages over time [47,68]. For smartphone apps utilizing check-ins, one study reported that, on average, participants showed relatively high engagement (86%), with all participants responding to at least one check-in [72], and some studies suggested that the addition of a clinician portal supports response rate [50,52,72]. However, one study showed a low response to check-ins towards the end of the study trial (13%) [54]. Studies examining the use of video-supervised OAT showed variable video response rates. These ranged from moderately low (31%) [64,71] to high (98%) [43] even when video uploads were compensated [64,71]. Interventions that included modules and homework found average access and completion rates of 27% [50], 41% [58], and 78–100% [60].

***Safety & privacy*:**Of the 34 included studies, 28 articles (82%) reported safety features of the technology. Regarding safety features, only six (21%) reported that the technology being examined was capable of providing additional crisis resources to support users in navigating elevated distress in real time (e.g., informational resources, recovery-related information, clinic phone numbers, support groups, etc.). Nine technologies (32%) reported the ability to connect users to real-time support through community message boards or clinician communication, via user-initiated access or risk-based alerts. Lastly, seven interventions (25%) included regular symptom check-ins to monitor user well-being. Only seven of 34 articles included safety indices (21%). Of the seven, four studies reported on serious adverse events/adverse events, with one study reporting no adverse outcomes [74]. The remaining three stated that the events were unrelated to the intervention [59,67,71].

Twenty (59%) studies reported on privacy components embedded within the investigated digital intervention. With respect to privacy considerations reported, only three of twenty reported training users on strategies to protect their personal health information (PHI) [68–70], four reported that the investigated digital tools were compliant with relevant privacy standards [43,64,66,67], five reported that PHI was stored on secured servers [43,44,67,69,74], and five reported the use of unique login and passwords known only to users [41,58,61,66,75]. Lastly, nine reported that the PHI entered into the technology was not accessible by healthcare professionals. Of note, none of the included interventions reported having undergone an external review of privacy features.

#### 3.1.2 Reported efficacy from included experimental trials.

Only five of the 11 included RCTs and one non-randomized study had primary efficacy outcomes. These trials are outlined in Table 3 below. Overall, the reported efficacy of these technologies is varied with respect to reduced use or abstinence from substance use. Preliminary clinical impacts, as explored in pilot trials, feasibility studies, and pilot RCTs, are examined in detail in the following section organized by the type of modality employed within the technological intervention.

**Table 3:** Included trials with primary efficacy outcomes.

First Author	Sample Size	Control	Intervention	Primary Outcome	*p*	Effect Size
**Randomized Trials**						
[41]	160 (80/group)	TAU	Therapeutic Educational System (TES): Internet-based community reinforcement approach.	Abstinence x Cognition	*Reported on factors influencing effectiveness. Lower cognitive scores predicted lower abstinence.*	
[44]	170 (92TES/79CM)	CM	Therapeutic Educational System (TES): Internet-based community reinforcement approach + Contingency Management (CM)	Abstinence (Longest Continuous (LC) & Total (T) in days)	LC *p* = .214 T *p*=.011*	5.5days 9.8days
[48]	14 (11Cog/3TAU)	TAU	Online Cognitive Training: 10 60-minute training sessions.	Pre-Post Cognition	*Showed pre-post improvements in various domains but did not conduct group comparisons due to discrepant sample sizes.*	
[71]	78 (39/group)	TAU	Video Directly-Observed Therapy (VDOT) for buprenorphine treatment.	Abstinence (% Negative UDS)	*p* = .07	RR = 0.78
[53]	41 21VDOT/20TAU)	TAU	Video Directly-Observed Therapy (VDOT) for buprenorphine treatment + Monetary Incentives.	Abstinence (% Negative UDS)	*p* = .816	OR=1.13
Non-Randomized Trials						
[45]	124 (62/group)	TAU	DynamiCare: a contingency management smartphone app.	Drug Use (abstinence)	*p*=<.05*	OR 4.31

Note: outcomes are between-group intervention outcomes. Feasibility outcomes, even if primary, have been collated in the feasibility section. * denotes significant group difference. TAU = treatment as usual, UDS = urine drug screen.

#### 3.1.3 Relapse prevention approaches.

One intervention focused specifically on relapse prevention. PIER1 is a text-messaging intervention for emergency room patients aimed at relapse prevention. The intervention sent text messages related to positive thinking, tailored coping based on craving severity assessments sent 2x/day, and feedback on daily opioid use and goal commitment at the end of each day. A pilot study (*n* = 20) was conducted. Participants were enrolled in 1-week increments with the ability to re-enroll for up to one month; however, this study reports on the first 7 days. The reported qualitative finding was that overall, participants provided positive feedback regarding the intervention, exemplifying themes of usability, self-empowerment, and social connection; however, they also desired a more personalized approach (i.e., desire to work with a person) [68]. Event-level data showed an increased risk for opioid use with higher craving severity. Over the first seven days, opioid use was reported 14 times, with 48 instances of cravings reported [68]. Given the pilot nature of the study, efficacy outcomes were not reported.

#### 3.1.4 Behavioural approaches.

***Contingency Management (CM)*:** Three interventions focused on contingency management approaches. DynamiCare, a CM smartphone app, was investigated in a non-randomized pilot study (*n* = 20) and an RCT (*n* = 124). The pilot trial investigated buprenorphine adherence, which was 76% throughout the trial, with a 72.5% submission rate of salivary toxicology tests and a 97% negative rate for substances [46]. Participant feedback suggests good ratings of likability, usability, and helpfulness (4.33-4.89/5) [46]. The RCT investigated UDS compliance and program attendance as the CM outcomes [45]. This study reported that individuals receiving the intervention were more likely to submit a urine test at times 3 (61–90 days; OR 4.31, *p* = < .05) and 4 (91–120 days; OR 9.43, *p* = < .05) and exhibited higher attendance rates across the time points (*M* = 20% higher) [45]. Submission of UDSs declined over time in both groups but remained higher in the intervention group [45].

PROCare Recovery is a micropayment-based rewards smartphone app targeting a range of recovery goals (e.g., medication/appointment adherence, completion of online educational modules) that was investigated in a multi-method pilot study (*n* = 10) [63]. Thematic analysis showed that participants liked micropayments (up to $150/month) and found the pre-determined payment blockage on the provided debit card (i.e., could not withdraw cash or spend at a liquor store or bar) highly acceptable. Reported usability ratings were also high (*M* = 92.2, range = 72.5 -100) [63].

TIES, a text-message CM intervention to promote adherence to supervised methadone appointments, was examined in a small-sample feasibility RCT design (*n* = 10), which reported a 96% concordance rate between the tech-user-reported and pharmacist-reported attendance rate, 77% text message delivery rate, and a close to 0% response rate, possibly due to concerns regarding the cost of text messages [59]. Of note, 50% of this small sample was experiencing homelessness at the time of data collection [59].

***Community Reinforcement Approach (CRA)*:** Three interventions focused on CRA approaches. A web-based intervention called Therapeutic Educational System (TES) was investigated in two RCTs. The initial RCT (n = 160) sought to characterize cognitive difficulties among individuals receiving methadone treatment and investigate whether a web-based intervention may mitigate cognitive effects [41]. This study found that more cognitive impairment scores (i.e., lower cognitive scores) predicted better study retention (η^2^ = 5.03, *p* < .05) but lower rates of continued abstinence. Further, lower scores on attention/mental control, memory, information processing accuracy, and general cognitive functioning predicted lower levels of overall abstinence but only in the TAU group, suggesting TES may support abstinence in individuals with cognitive impairment [41]. Another RCT (*n* = 62) found that compared to TAU, the intervention group showed significantly better opioid abstinence overall (i.e., 48% vs. 37% *F*(1, 158) = 5.90, *p* = < .05) and via UDS (i.e., 59% vs. 43% *F*(1, 158) =8.81, *p* =<.01). Participants did not significantly differ with respect to their counselling attendance. Participants in the intervention group completed a mean of 27.56 modules (SD = 24.44) each lasting an average of 26.86 minutes [58]. Rated on a 100-point scale, participant feedback indicated that TES as likeable (*M* = 75.6), useful (*M* = 77.5), usable (*M* = 80.7), and contained new information (*M* = 74.8) [50]. Qualitative feedback demonstrated the use of the intervention in high-risk situations for substance use and to manage cravings [50]. Participants also reported barriers to using the TES intervention, including technical barriers (i.e., small screen/touch buttons, remembering how to log in) and finding it boring. The main feedback on improvements was to broaden TES topics to substances other than opioids and related mental health experiences (i.e., self-esteem, anger, anxiety, drawbacks of substance use, etc.) [50]. reSET-O is a commercially available prescription digital therapeutic supported by PEAR therapeutics that delivers content based on TES through a smartphone app and clinical provider portal [54]. A pilot study (*n* = 15) investigating reSET-O had a retention rate of 47%, and 33% engaged with the intervention continuously, even with the use of monetary contingency management for module completion and feedback that reSET-O was generally liked (i.e., easy to use, safe space, enjoyable) [54]. The average amount paid to participants for module completion was $96.00 USD, and they completed an average of 15 modules. This study reported that cravings reduced over the course of the intervention from 53% to 13% [54].

Christensen et al. (2014) compared CM to CRA. Participants in the CRA group exhibited more total days of abstinence (*M* = 9.7 days) than those in the CM group. Having received prior treatment seems to have bolstered abstinence in the CRA group. The CRA group also showed a reduced hazard of dropping out of treatment (hazard ratio 0.47; 95% CI [0.26, 0.85]) [44].

***Video-Directly Observed Therapy (VDOT)*:** Several studies (*N* = 6) examined VDOT, which is take-home OAT medication (i.e., methadone or buprenorphine) with video-monitored adherence. Participants are asked to video themselves via personal or loaned smartphones or webcams, taking the medication and upload it to a clinician server asynchronously. One RCT (*n* = 78) examined the efficacy of VDOT and found that the control arm (TAU) showed significantly better abstinence from opioids (64% (95% CI: 55–74%) vs. 50% (95% CI: 40–63%); RR = 0.78 (95% CI: 0.60–1.02, *p* = 0.07)). and treatment engagement (82% (95% CI: 71–95%) vs. 69% (95% CI: 56–86%) respectively; RR = 0.84 (95% CI: 0.65–1.10, *p* = 0.20)) compared to the group that received VDOT due to limited intervention engagement [71]. Another RCT examined incentivized VDOT, and while VDOT participants were more likely to commence treatment (71.4% versus 30.0% respectively; OR [95% CI]: 6.24 [1.46-26.72], *p* = .014), continued opioid use and non-adherence were comparable across groups [53].

Regarding pilot feasibility studies, included studies reported varying video submission rates: 72% (*n* = 41) [49], 98% (*n* = 58) [43], and 31% (*n* = 39) [64]. Younger age (under 40) and once-daily dosing were positively associated with video submission, whereas non-white race, less than high school education, history of previous buprenorphine treatment, and three or more treatment attempts were negatively associated [64]. Another pilot study reported 10% of medication non-adherence [43]. Associated benefits with VDOT were reduced weekly travel time and cost by 86% (median cost saved was $72.00, median time saved was 5.5 hrs), and 98% of participants were in treatment 12 months later [43]. Qualitative feedback from participants was that they liked the accountability but felt discomfort viewing themselves in the videos [49].

***Motivational Recovery Coaching (MRC)*:** One intervention used an MRC approach. MySafeRx is a multi-technology intervention combining behavioural regulation techniques (i.e., use of an electronic pill dispenser) and MRC via text messaging and video conferencing through a smartphone app. Each day, the participants would meet with an MRC coach who provided coaching and a code for their pill dispenser so they could receive their buprenorphine. A small open pilot study (*n* = 12) reported on study feasibility; the authors reported a 66% study retention rate, and participants met with the MRC coach an average of 72% of days in the intervention [67]. Further, 90% of participants successfully used the intervention successfully after the training session [67]. Regarding participant feedback, overall satisfaction was rated well (i.e., 4.3/5 ± 0.7), and nine of the twelve participants reported a desire to continue in the intervention [67].

***Reminder-based interventions***: One intervention investigated in a feasibility survey study (*n* = 93) examined the use of text-messaging reminders to enhance appointment attendance for an in-office buprenorphine program [69]. Results indicated that 97% of participants reported that text message reminders helped them adhere to scheduled appointments, and that text messaging (91%) was favoured over phone (3%) and email reminders (6%). The most common barriers to attendance were transportation difficulties (34%) and time off from work/school (31%). However, this study did not measure or report on potential efficacy (i.e., no attendance rates were reported).

#### 3.1.5 Cognitive behavioural and cognitive approaches.

***Cognitive Behavioural Therapy (CBT)*.** Two interventions examined technology-delivered CBT-informed approaches to support various aspects of OUD. Two studies investigated the use of Recovery Line (a voice/call-activated computer-based CBT program). Compared to TAU, a pilot RCT (*n* = 36) found that individuals receiving Recovery Line were more likely to remain abstinent on the days they accessed the program; however, it did not find overall group differences [60]. Following this trial, two additional RCTs, published together, examined augmented means of delivery to improve intervention engagement [61]. The first was to have personalized recommended modules based on an intake call, compared to others using Recovery Line without recommended modules (*n* = 60). Apart from participant perspectives on ease of use, no significant improvements were found via the recommendations. However, the trial did report that abstinence from opioids and perceived coping abilities improved for both groups over the course of the intervention [61]. The second was augmented with text-message reminders, and groups were short, medium, and long-latency text messages. Short-latency messages were initially found to produce greater engagement in the intervention, especially among men, but this effect diminished over time [61].

One study (*n* = 50) examined an adjunctive text messaging program to in-person OAT treatment, with three functions: patient-provider communication, adherence reminders, and self-management resources informed by CBT and motivational interviewing (MI) [70]. The overall response rate was 12%, with 88% of participants responding to at least one text and a mean of eight responses per participant. Out of the responses, the most common topics were replies to CBT content and rescheduling appointments. Three participants requested to stop receiving text messages. The study did not report on efficacy items.

***Illness Management (IM)*.** One intervention focused on multifaceted illness management. Two studies examined the HOPE (Heal. Overcome. Persist. Endure.) app, which is an adjunctive app to medication-assisted therapy and includes various lived-experience co-designed features that appear to be CBT-informed (i.e., symptom and medication tracking, trigger/craving tracking, positive data logs for encouragement) paired with a peer-to-peer support feed [52,72]. A feasibility pilot (*n* = 25) found the HOPE app to be feasible, reporting high usability ratings and positive qualitative feedback (i.e., participants valued the self-monitoring aspects and improved access to resources, support, and care) [72]. In a secondary analysis of the same sample, retention at month six was 56%, with 88% using the provider messaging and 100% using the daily check-in at least once in the past month [52]. Over half continued to use the provider messaging, and 76% continued to use the daily check-ins throughout the six-month intervention period [52]. Regarding efficacy outcomes, 32% of participants tested positive one or more times throughout the intervention, with an average time to opioid use of 1.98 months (SD 1.19). Self-rated self-efficacy scores were significantly higher post-treatment [52].

***Cognitive training*.** One intervention examined remote cognitive training. A small initial efficacy pilot trial (n = 14 males) examined a virtual reality cognitive training program on cognitive impairment in individuals recovering from OUD [48]. This study reports several significant pre-post results across various cognitive domains, including executive functioning, verbal memory, sustained attention, and some aspects of cognitive flexibility and decision-making [48], but RCTs are required to replicate effectiveness.

### 3.2 Commercial review results

Our search identified 21 unique technological interventions, outlined in Table 4. Thirteen (61%) were smartphone applications, six (28%) were wearable technologies, and three (14%) were medical devices, including an opioid detector, a medication dispenser, and mobile brain-sensing platform. Of the 21 technological interventions, eight (38%) have conducted research trials or are in the process of doing so to test their efficacy. However, these trials were either pilot studies or in the early development stages. The majority are available only in Canada and the United States. However, only two technologies were developed in Europe (i.e., CLM-0P01, Closed Loop Medicine Ltd, 2023 and Prapela Bassinet Pad, 2023). Thirteen smartphone applications were available for individuals with opioid use disorder. While the structure of these applications varied, the apps generally aimed to prioritize user safety, supplement treatment, and promote recovery by providing access to resources and real-time support. Three digital health platforms include an opioid detector, a smart key for a medication dispenser, and a mobile brain-sensing platform. However, no information exists on whether these technologies have FDA approval.

**Table 4:** Commercially available technology for OUD.

Technology Name	Technology Type	Function	Approved by Healthcare Body	Supported by Research
**Brave**	Application	Users set up an overdose plan and will be connected with someone who can send help while using drugs alone.	No	No
**OpiSafe**	Application	An app for practitioners to access a summary of patient information to better manage opioid use. A patient version of the app, OpiRescue, allows the patients support network to locate information should they ever have an overdose.	No	No
**reSET-O (Pear Therapeutics)**	Application	An app intended to increase retention of patients in outpatient treatment by providing CBT as an adjunct to outpatient treatment.	Yes	Yes
**DCH-003 (OUD) (DynamiCare Health)**	Application	An app that rewards healthy behaviours, includes a coach to help set achievable goals. helps user to schedule and follow through recovery meetings. Also includes 90 self-paced units to build recovery skills.	No	Yes
**A-CHESS**	Application	An app that provides antecedent-appropriate intervention to boost autonomy through multiple tools.	No	No (Pilot in progress)
**NSS-2 Bridge**	Medical Device	Stimulator that is placed behind the patient’s ear that emits electrical pulses to stimulate branches of certain cranial nerves to provide relief from opioid withdrawal symptoms.	Yes	Yes
**Prapela Bassinet Pad**	Medical Device	Vibrating bassinet pad to calm opioid exposed newborns to help them breathe, relax and sleep.	Yes	Yes
**OPI-Wipes**	Detector	Polymer based wipe that can detect traces of opiate residue, giving first responders at the scene of an overdose an indication of the presence of an opioid in the vicinity.	No	No
**COR-12 for Opioid Addiction**	Application	An app that is part of a community treatment program that uses medication assisted treatment.	No	No
**My Opioid Manager**	Application	An app that is an educational and informational resource to help patients suffering from chronic pain understand and manage their pain with OUD.	No	No
**BupreCare System (MedicaSafe Inc)**	Medication Dispensing Device	Medication pre-packaged in secure cartridges that are dispensed by a programmable SmartKey, a device that authenticates the patient and provides access to the right dose at the right time	No	No
**CLM-0P01 (Closed Loop Medicine Ltd)**	Wearable Medical Device and Platform	A medical device platform to coach, support and guide each patient and discover insights to improve care, and it collects data through wearable devices to fully support disease management and recovery.	No	No
**Digital Intervention System: Opioid Addiction Relapse (DORS) (Behaviour LLC)**	Application	An app that will help protect a patient if they are using alone. If there is no engagement with the app while using, the STARS emergency response crisis will call to determine if the patient needs additional support.	No	No
**Dual Sensor Regional Oximeter (Ayuda Medical LLC)**	Wearable Medical Device and Application	Discreet Wearable device that will detect signs of overdose and if necessary, inject a rescue dose of naloxone. Comes with a mobile app that will contact patients within 24 hours of overdose, provide them with treatment options and support them during recovery.	No	No
**Hope Band (Hashtag)**	Wearable Medical Device and Application	A wrist mounted oximetry device that is paired with a smartphone device. Device detects oxygen levels, sends out messages to app and contacts in case of overdose.	No	No
**iPill Dispenser Mobile App (ipill Smart Dispenser)**	Medical Device and Application	An app that uses a biometric authentication process to operate a portable tamper-resistant device to ensure patient treatment for pain, prevent overconsumption and diversion of opioids.	Pending	No (unpublished pilot studies)
**Modia (Orexo AB)**	Application	Automated Web-based software program that uses MI techniques. The MODIA program comprises 24 modules or “chats” that patients are instructed to work through independently. Patient responses dictate subsequent content, creating a “simulated dialogue” experience between the patient and program.	Unclear	No (research trial in progress)
**NeuromarkR Platform (Neurotype Inc)**	Mobile Brain sensing platform	NeuromarkR™ quantifies brain physiological responses to possible triggers for people with Substance Use Disorder. Provides clinicians and patients with tools to objectively detect triggers for relapse such as craving or distress at the point of care.	No	No
**OARS +CM (Q2i)**	Application	Mobile app that uses Contingency management to improve treatment initiation and retention, improve engagement and adherence to treatment plans, reduce missed appointments, and reduce Emergency Department readmissions.	No	No (trials in progress)
**Second Chance (SoundLife Inc)**	Application	Converts a smartphone into a short-range active sonar system capable of monitoring breathing and detecting overdose.	No	No
**Wearable Biometric Device (OpiAID LLC)**	Wearable Medical Device	A wearable biometric device platform that will predict use and withdrawal in those undergoing medication-assisted therapy for opioid use disorder.	No	No

*Note: Information is accurate at the time of the initial search. Information may have changed since, given the rapid pace of commercialization.*

Additional research was identified in the commercial review that was not in the academic review. These studies may not have been identified in the academic review for several reasons, including that they may not have been indexed by the databases we utilized, or may not have met criteria for the academic review and were screened out (i.e., brief reports, no a priori research question, not being cited in included studies, conducted on technology that did not leverage personal devices, etc.). We included technologies beyond those that leverage personal devices to provide a broad understanding of the types of technology available in the field; however, going forward, this manuscript focuses on those that do leverage personal devices to enable better synthesis of information between the two reviews. To understand the link between commercialization and academic research, we examined the funding sources of studies identified in both reviews presented in Table 5.

**Table 5:** Funding sources and conflict of interest statements focusing on personal technologies.

(Technology) First Author	Funding Source	Conflict of Interest (COI) Statement
**Studies included in the scoping review**		
(TES) Acosta, 2012	National Institute on Drug Abuse	Supervising author affiliated with developer. Explicit statement that COI is mitigated with institutional support.
Marsch, 2014	National Institute on Drug Abuse	First author is affiliated with developer and reported working extensively with institutions to mitigate COI. Authors state the funder has no role in the study.
Guarino, 2016	National Institute on Drug Abuse	Supervising author is affiliated with developer and reported working extensively with institutions to mitigate COI. Authors state the funder has no role in the study.
Bosse, 2022	National Institute for Drug Abuse	First author and three other authors report being employed by developer and another a consultant.
(NN, CRA vs. CM) Christensen, 2014	National Institute on Drug Abuse and the Wilbur Mills Endowment.	Two authors are affiliated with the developer with an explicit statement of institutional COI mitigation. Analyses were designed and conducted by a third-party statistician.
(HOPE app) Hodges, 2021	Virginia Department of Health and the University of Virginia HEAL Grant	Three authors report consulting agreements with The developer.
Waselewski, 2021	Virginia Department of Health and the University of Virginia HEAL Grant.	Five authors report consulting agreements with the developer
Flickinger, 2022	Virginia Department of Health and the University of Virginia Helping to End Addiction Long-term (HEAL) Grant	Three authors report consulting role with the developer. For all other authors, there are no declarations. Authors state the funder has no role in the study.
(NN, Cognitive Remediation) Gamito, 2017	NR	NR
(VDOT) Godersky, 2020	National Institute on Drug Abuse – research and business partnership grant. Company: emocha	One author reported receiving commercial royalties and served on a different advisory board. Another author reported holding a separate grant with the developer. All other authors report no conflicts. Authors state the funder has no role in the study.
Schramm, 2020	National Institute on Drug Abuse research and business partnership grant. Company: emocha	One author declared advisory relationship with different biomedical company. The remaining authors declare no competing interests.
Tsui, 2021	National Institute on Drug Abuse research and business partnership grant. Company: emocha	One author declared advisory relationship with different biomedical company. The remaining authors declare no competing interests.
Holtyn, 2021	National Institute on Drug Abuse and Centers for Disease Control	Institutional stakeholder interest and royalties/fees. Reviewed by institution and found to be in accordance with COI policy.
Brooklyn, 2022	Substance Abuse and Mental Health Service Administration Medication Assisted Treatment for Prescription Drug and Opioid Addiction (SAMHSA MAT-PDOA) and Vermont Department of Health.	First author reported being a consultant for the developer.
Radick, 2023	National Institute on Drug Abuse research and business partnership grant. Company: emocha	None declared.
(A-CHESS) Gustafson, 2016	National Institute on Drug Abuse	Four authors report a shareholder interest in the technology. Authors report working extensively with the institution to mitigate COI. Authors state the funder has no role in the study. All other authors report no COI.
(iCOPE) Langdon, 2020	National Institute on Drug Abuse	None declared.
Langdon, 2021	National Institute on Drug Abuse	None declared.
Langdon, 2022	National Institute on Drug Abuse	One author reported an agreement with developer for another trial.
(TIES) Metrebian, 2021	National Institute for Health Research	Authors report preexisting relationships with various policy bodies and companies via consultation fees. Authors state that the research presented was independent.
(Recovery Line) Moore, B., 2013	National Institute on Drug Abuse and the State of Connecticut, Department of Mental Health and Addiction Services	None declared.
Moore, B., 2017	National Institute on Drug Abuse	Authors state the funder has no role in the study.
(Pop4Teens) Moore, S., 2019	National Institute on Drug Abuse	One author reports affiliation with developer.
(PROCare Recovery) Proctor, 2022	National Institute on Drug Abuse	Two authors report additional NIDA grants to study the technology. Another author is the president of the developer company.
(Marigold App) Scherzer, 2020	National Institutes of Health research and business partnership grant. Company: Marigold Health	Two authors report being employed by the developer.
(MySafeRx) Schulman-Oliver, 2018	National Institute on Drug Abuse and MedicaSafe	None declared.
(PIER1) Suffoletto, 2017	Emergency Medicine Foundation and National Institute of Drug Abuse	None declared.
(NN, SMS Reminder) Tofighi, 2017	Research in Addiction Medicine Scholars Program	None declared.
(TeMeS, SMS Reminder) Tofighi, 2022	National Institute on Drug Abuse	None declared.
(NN, Attention Bias Training) Zhang, 2018	NAMS**	None declared.
Zhang, 2019	Singapore Ministry of Health’s National Medical Research Council	None declared.
**Included in Both Scoping and Commercial Review**		
(DynamiCare) DeFulio, 2021	Ohio Opioid Technology Challenge awarded to DynamiCare Health Inc., and BrightView Health, Inc.	First author and another author report consulting agreements and another is employed by the funding partner. The remaining author declares no conflict. Authors state the funder has no role in the study.
DeFulio, 2023	National Institute on Drug Abuse	The first author reports consulting agreement the developer. The remaining authors declare no conflicts. Authors state the funder has no role in the study.
(reSET-O) Kawasaki, 2022	National Center for Complementary and Integrative Health.	None declared.
**Included in the Commercial Review**		
(reSET-O, continued) Maricich, 2022 Valez, 2021a Valez, 2021b Wang, 2021	Pear Therapeutics was the funder and developer. Pear Therapeutics was the funder and developer. Pear Therapeutics was the funder and developer. This study was funded by Sandoz, a Novartis Division. *Which had a partnership agreement with Pare Therapeutics [76].	Authors state that all authors are either employees or contractors for the developer. Additionally, peer reviewers on this manuscript were reported to have received an honorarium from the journal for their review work. One author is an employee of the developer, and all other contributing authors reported consulting contracts with the developer. Authors state they did not receive honorarium for manuscript preparation. They additionally state “*The authors have no relevant affiliations or financial involvement with any organization or entity with a financial interest in or financial conflict with the subject matter or materials discussed in the manuscript. This includes employment, consultancies, honoraria, stock ownership or options, expert testimony, grants or patents received or pending, or royalties.”* Two authors report employment by the developer, with a third reporting a consulting agreement. The same italicised statement above is included in the transparency statement for this paper. Two authors are employees of Sandoz, and another reported a consulting agreement. Additionally, peer reviewers on this manuscript were reported to have received an honorarium from the journal for their review work.

** = Acronym not explained, NR = Not Reported, NN = No Name reported in the included paper.

While all the research identified in the academic review was funded by public grant agencies, it was overwhelmingly common across both review methods for researchers to have financial relationships with the tech companies developing the technologies, either through formal employment or consulting contracts. In most cases, these potential conflicts of interest (COIs) were reported as institutionally mitigated to ensure independent research and to clearly state that the funder or developer did not have a role in the study or in the use of third-party staff, such as analysts and biostatisticians. In the commercial review, an interesting case study regarding funding and COI emerged regarding reSET-O, a smartphone application developed by Pare Therapeutics. Four studies, in addition to the one identified in the academic review [54], examined reSET-O. Each of these studies was funded by Pear Therapeutics or a partner company, and the majority of authors reported financial relationships with the company. In two of the four studies [77,78], the authors stated they had no relevant financial interests related to the manuscript, while also listing financial partnerships, and the other two studies [79,80] reported financial partnerships and that the reviewers received honoraria for their roles as reviewers provided by the journal. All four studies report positive outcomes following the use of reSET-O and present net budgetary savings.

## 4. Discussion

This review was undertaken to describe and summarize the current state of the commercialization of and research examining digital health tools that leverage personal devices to support recovery outcomes for individuals seeking treatment for opioid use. Overall, compared to other areas of mental health and substance use, there was a relatively small number of studies included in this scoping review, with other reviews examining digital health in substance use broadly reporting only 9% of studies examined opioid misuse [81]. Despite a smaller number of studies, overarching patterns of findings are consistent with reviews of digital mental health in other populations, such as schizophrenia [82], bipolar disorder [83], OCD [84], and the broader substance use context [81]. Like other areas of digital mental health research, the use of digital tools for individuals with OUD seems to be feasible and highly acceptable and small pilot trials exhibit promising outcomes.

However, as in other reviews on opioid use [33] and beyond, there is mixed evidence from primary efficacy RCTs, with no intervention or group of interventions being a clear frontrunner. Given the preliminary state of research in this area, it raises questions regarding the number of technologies for OUD that have received FDA approvals as therapeutics. Further, this highlights a key concern regarding the number of technologies (*n* = 17/21, 81%) available commercially with no scientific review (i.e., no FDA approval and no published peer-reviewed research at the time of the present analysis). This finding also echoes concerns in other areas of mental health regarding the state of commercially available technologies marketed to support recovery without evidence [85].

Another cross-cutting finding reiterated in our review is the potential impact of varying engagement on effectiveness outcomes [86]. As seen across the field [87], engagement with digital tools in the included studies tends to decrease over time, leading to varying levels of intervention participation, resource utilization, and study retention, all of which may impact effectiveness metrics. Further, engagement metrics in this review and across the field are heterogeneous, non-standardized, and lack clarity on clinically meaningful participation [88,89]. As other studies have highlighted, a key factor in technology use and engagement is user and infrastructure fit [90–92]. This ties into an ongoing debate on the role and function of technology in the treatment of substance use disorders [93] and other mental health conditions. As in other areas of the field, the optimal role of technology is obscured by a lack of understanding of the relationship between engagement and efficacy, and by limited discussion in published manuscripts of the nesting infrastructure in which the technology is deployed, hindering understanding of external-to-participant factors that may impact future implementation. Emphasis is being placed on environments and infrastructure as essential, and digital health tools are being conceptualized as elements of complex interventions to be blended into existing care structures (rather than stand-alone). Many interventions utilize multiple technologies, underscoring the complexity of these tools and highlighting the need for adequate implementation planning.

Similarly, our review highlights a dearth of information on critical implementation factors such as privacy and safety. As in other areas of mental health, a lack of transparency or understanding of associated privacy and safety features/limitations has been identified as a barrier to both client and clinician adoption [81,94]. More specific to substance use treatment are considerable ethical considerations related to privacy and safety. First, in many places, personal consumption of illicit substances is still illegal, creating a potentially serious legal risk for technology users [81], an area with complex laws and policies and very little clear guidance. Thus, a clear determination is required regarding whether data collected within these technologies is protected as health information or whether this information may be subject to a subpoena from a court of law. This also highlights the need for clear, accurate, and transparent user privacy agreements that include this information.

Second, there are complex medical-legal responsibilities for practitioners to navigate when using these technologies, in which client information is visible, including mandatory reporting [81]. For instance, in Canada, medical doctors have a duty to report impaired driving. If a client were to log substance use while driving in the technology, and this information were seen by their doctor, would this be reportable? If so, it would be a barrier to clients’ use of the technology, may undermine trust in the technology, and damage rapport. Another key concern is navigating the use of technology as a communication facilitator and the related medical-legal responsibilities of the provider and the technology developer in a crisis situation. There are concerns regarding clinician availability viatechnology during a crisis, whether this may damage rapport or lead to serious harm (i.e., outside of working hours or with other clients).

Additionally, there are concerns regarding the appropriate resourcing of apps, the determination of responsibility, and what happens if an app developer fails to provide adequate crisis resources, whether through initial design or failure to maintain working crisis features [95]. These concerns are critical to clinician adoption and client safety, especially in a population where associated mortality is a significant risk, like OUD populations. Based on the reported information, it would seem that the use of digital therapeutics is generally safe in OUD populations; however, without additional data, a definitive evaluation is not possible. Thus, future research should ensure that these areas are adequately addressed in published manuscripts to aid the understanding of how to mitigate the above risks. Furthermore, future research should consider safety reporting beyond SAEs and AEs, which, in this context, can be very difficult to attribute to factors such as digital technology use, to enhance our understanding of user risk. Worsening of symptoms, increased substance use and associated risk (i.e., using in more or less risky situations), and whether the app itself caused any distress or frustration and its outcomes (i.e., did the app malfunction or provide harmful information leading to distress, substance use, impulsive behaviours, etc.) should also be considered.

Specific to OUD, most included studies that examined efficacy focused on primary outcomes of abstinence or medication adherence. While clearly important, these variables are only part of the recovery picture. More studies are required to examine holistic recovery indicators, such as the severity of cravings, self-efficacy, perceived coping, increased symptom insight, and improved recreation and social relationships. Further, abstinence may not always be the individual’s goal (e.g., harm reduction approaches) and therefore may not be the most appropriate indicator of intervention effectiveness. Other reviews have found that technologies targeting opioid misuse often target pain management rather than OUD itself, which is a multifaceted and complex mental health condition [93]. Thus, future development in this area must be responsive to the needs of individuals with OUD and reflect the multiple care pathways that exist.

Lastly, most samples in the included studies are derived from white male populations, which has implications for the generalizability of findings and health equity, given the significant structural inequities faced by underrepresented racial and gender populations. Racialized individuals are less likely to receive referrals for OUD treatment, be treated with OAT, and complete treatment [96–98], which is reflected in the lack of diverse research samples. Research postulates that this may be in part owing to a preference for racial minority groups to receive non-OAT treatment for OUD [99], calling for more options for identity-congruent and culturally informed care. Given that the majority of studies included in this review also focused on adjunctive support for OAT adherence, the same need is reflected in digital health interventions.

Further, studies have shown that across OUD treatment research, women are vastly underrepresented [100], despite some evidence that opioid use may be higher for young women [101,102] and that gender-specific resources may be helpful [103]. Further, despite housing insecurity being reported in a number of studies as a factor influencing the feasibility of interventions, only six studies reported on the proportion of the sample that were experiencing homelessness, no fixed address, or unstable housing. This gap has implications for employing digital health interventions in contexts where housing instability and homelessness are common. Additional implementation considerations, such as access to devices and data, will need to be addressed to ensure that health and service access disparities are not compounded.

Beyond future research directions, this data also has important implications for regulatory considerations. First, it calls into question how mental health apps are regulated and whether mental health applications are appropriately scrutinized before public availability. The majority of apps and wearable technologies identified in our commercial review were not supported by peer-reviewed research and were not approved by a health regulatory body, yet they claim to support individuals seeking support for opioid use. In a context of the pronounced and acute health risks associated with opioid addiction, a higher degree of scrutiny and regulation is likely needed. In many countries, including Canada, the United States, and parts of Europe, this is generally related to how regulatory bodies classify mental health apps and how developers market and define their products. Only mental health apps and other personal devices that meet the definition of a medical device (i.e., a device intended for diagnosis or treatment) and have a risk rating higher than “low risk” are subject to regulatory body approval [104–106]. However, by primarily focusing on the intended use, these regulatory procedures neglect the user context and fail to account for the inherently different risk levels associated with different target populations, regardless of whether the intended use is clinician- or wellness-oriented. Thus, if a company frames the intended use of the product as wellness support rather than clinical use, it may be able to bypass regulatory scrutiny.

We recommend an expansion of the definition of medical device criteria to include use in clinical populations, regardless of intended use. The distinction between wellness and clinical use can then be differentiated in the risk evaluation, where wellness use may be assigned a “low risk” rating if appropriate, based on the target population. While not claiming to be a treatment, many wellness apps claim to help people with mental health-associated experiences and symptoms like cravings, mood tracking, being more active, eating behaviours, or addressing stress, which can increase risk in higher-risk populations. Therefore, more inclusive definitions of medical devices are likely needed to ensure rigorous review of mental health apps within systems that can also support access to potentially helpful apps and devices. An example of this is the Digital Healthcare Act in Germany, which supported a fast-track pathway for mental health apps to become certified, prescribable, and publicly funded once security and evidence standards were met. While this approach still utilizes medical device criteria, it appears to include technologies classified as “low risk” [107].

Further, the results presented in this review regarding funding and COI offer an opportunity to explore the ethical navigation of financial relationships and the use of academic research to enhance market credibility. While most authors clearly state that they worked with their institutions to mitigate any potential COI as recommended by ethical standards in research, the prevalence of financial partnerships in this field is noteworthy. Specifically, the development process for digital tools, like mental health apps, falls somewhere between psychosocial interventions and medications. The risk profile of apps is more similar to that of psychosocial interventions; however, their potential for and reliance on commercialization are more comparable to those of medication. Like in medication development, commercialization is an eventual necessity for apps. App development is time-consuming and costly, and most public grants provide only initial development funding (i.e., seed funding), leaving funds scarce for engagement-driven graphic design, long-term maintenance, or technology updates [108]. Commercial funding can help fill this gap. Commercialization can also lead to collaboration with multidisciplinary teams beyond academia, potentially facilitating the development of improved digital health products [109]. For these reasons, industry partnerships are considered a necessity for the future of this field to ensure adequate technological support, scale, and accessibility [108].

Further, it’s been proposed that academic-industry partnerships can leverage each other’s strengths to improve digital health tools. Where industry is often described as having limited clinical utility and favouring profit over efficacy, academia demands rigour, is evidence-based, ethical, and includes clinical populations industry often can’t reach [109]. Where academia is often slow, underfunded, and underdisseminated, industry thrives in a fast-paced environment, with commercialization and funding at the forefront [109]. However, these differing strengths and institutional cultures can also lead to points of friction in a relationship, and therefore require careful navigation to ensure safety, clinical utility, and privacy are prioritized. Currently, there are no regulatory or policy guidelines specific to navigating academia-industry partnerships for digital health research that provide recommendations for balancing competing pressures among clinical utility, safety, and profit while mitigating dual relationships and potential COIs. Hall and colleagues (2025), have developed the Principles of Industry-Academic Partnerships (PIP) guidance document. Particularly relevant to this topic is the recommendation to hold a Ways of Working (WOW) Workshop early in the partnership to facilitate transparency, align expectations and priorities, and gauge each other’s risk appetite (i.e., willingness to take risks and what those risks entail) to inform the working relationship [110].

Similarly, this review highlights important factors to aid clinicians’ decision-making if considering adding digital mental health tools to their practice. First, given the paucity of research-supported digital health tools presented in this commercial review, it is important for clinicians considering a specific tool to ascertain whether it has been properly evaluated in terms of privacy (i.e., has it undergone a privacy evaluation which determined it meets legal standards for health data in your country), safety (i.e., what safety measures are in place, does it comply with your professional and institutional standards), and efficacy (i.e., have there been any large RCTs suggesting there is an added benefit). Other considerations for clinicians mirror future directions for research, including considering how the digital tool will fit into the clinical context, compatibility with existing technological infrastructure, impact on workflow, clinician usability, as well as contextual factors that impact usefulness in specific populations (i.e., minority groups, insecurely housed people) or treatment approaches (i.e., most focus on abstinence). More detailed resources for evaluating whether a digital tool is right for your practice have been developed by organizations like the American Psychiatric Association [111] and the Mental Health Commission of Canada [112], including lists of evaluated apps.

### 4.1 Limitations

The results of this review should be considered within the context of methodological limitations. First, we did not consult a librarian when constructing search terms, though the search was modelled on a similar study that did. Regardless, this may have affected the breadth of studies identified. Relatedly, our search identified studies that were predominantly from the United States; while this is consistent with our commercial review, research and products from other countries may have been missed. However, we believe we have identified a reasonable representation of the existing research and products presented in this review, enabling an accurate, overarching description of the landscape. Second, there were insufficient RCTs examining consistent technologies and outcomes to conduct a rigorous meta-analysis; therefore, the results presented are descriptive aggregates rather than quantitative comparisons. Further, given the heterogeneous methods used to measure and report engagement and effectiveness outcomes, data on the overall quality and effectiveness of interventions are difficult to summarize and assess. Third, the information accessed through commercial channels is neither peer-reviewed nor verified in any standardized way. Thus, the quality of available products cannot be assessed.

### 4.2 Conclusions

Digital health in the area of opioid use is best characterized as nascent, an observation that is arguably at odds with the large-scale investments in opioid response and the populations affected. Many design and evidence-generation challenges remain, as do questions about the development of tools that will equitably and effectively serve diverse populations. For this field to advance and begin to have a substantial impact, more rigorous data are needed, and, equally important, more co-design and implementation science activities are needed to increase the uptake of digital health approaches across care contexts struggling to address the opioid crisis.

## Supporting information

S1 Text Figures S1-S3: Databases powered by OVID, expanded search terms. (DOCX)

S1 Fig PsychINFO search terms. (TIFF)

S2 Fig MEDLINE search terms. (TIFF)

S3 Fig Embase search terms. (TIFF)

S1 Checklist PRISMA checklist. (DOCX)
