# Supplementary material for: Digital mental health tools for use by individuals in opioid use recovery: An academic scoping and commercial review
Source: PLOS Digit Health. 2026 Jul 30;5(7):e0001544. doi: 10.1371/journal.pdig.0001544 (PMC13422840; doi:10.1371/journal.pdig.0001544)
Supplement: S1 Text — (DOCX) [file pdig.0001544.s001.docx]

**Supporting Information: Databases Powered by OVID, Expanded Search Terms**


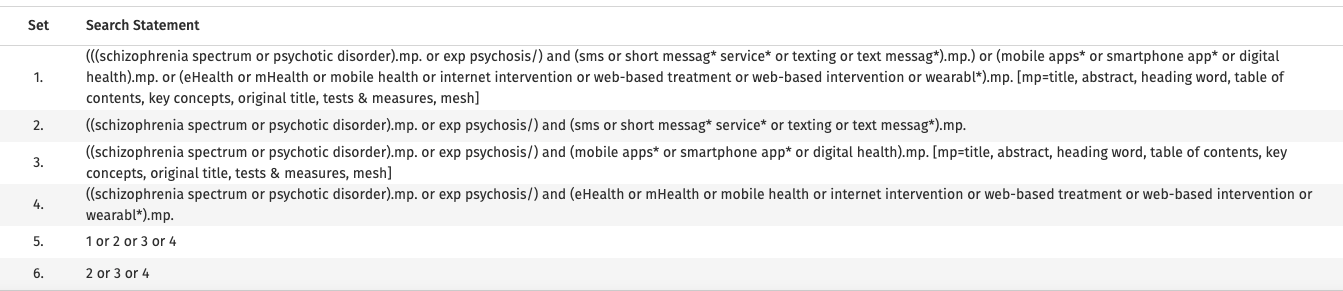


Fig S1 PsychInfo Search Terms


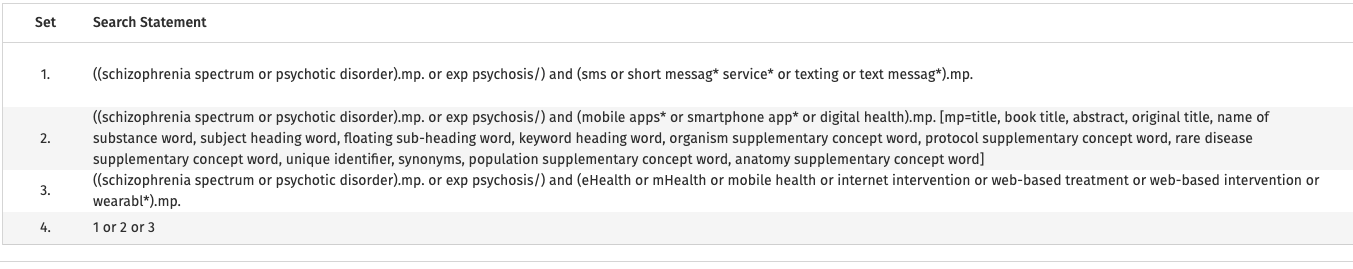


Fig S2 MEDLINE Search Terms


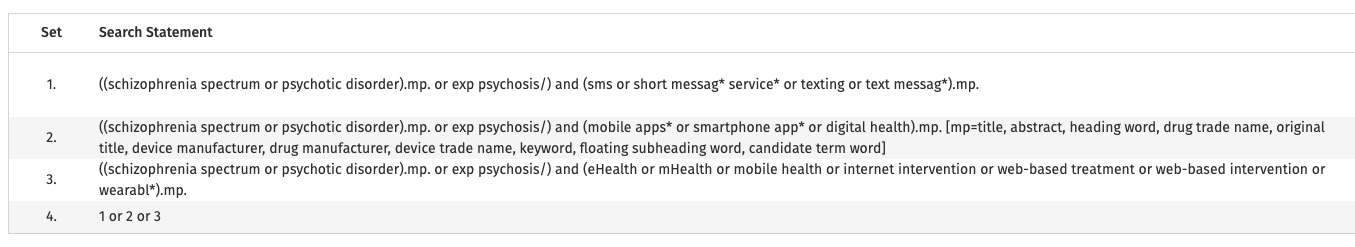


Fig S3 Embase Search Terms
