# Supplementary figures and images for: Digital mental health tools for use by individuals in opioid use recovery: An academic scoping and commercial review
Source: PLOS Digit Health. 2026 Jul 30;5(7):e0001544. doi: 10.1371/journal.pdig.0001544 (PMC13422840; doi:10.1371/journal.pdig.0001544)

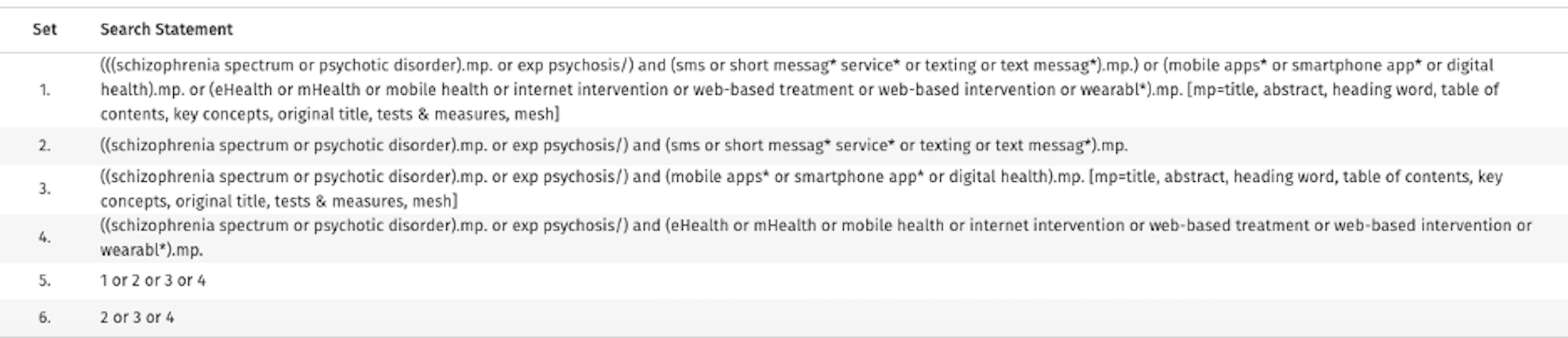

Supplement: S1 Fig — (TIFF) [file pdig.0001544.s002.tiff]

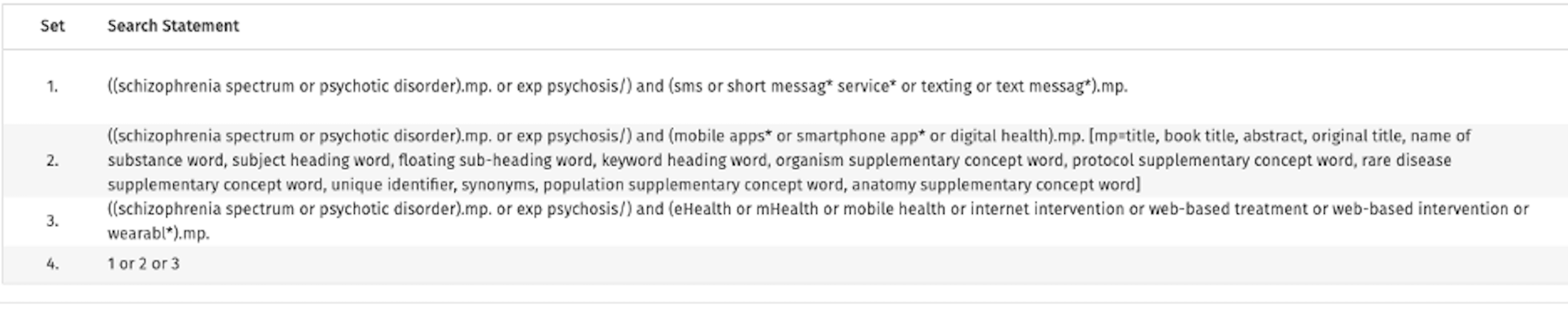

Supplement: S2 Fig — (TIFF) [file pdig.0001544.s003.tiff]

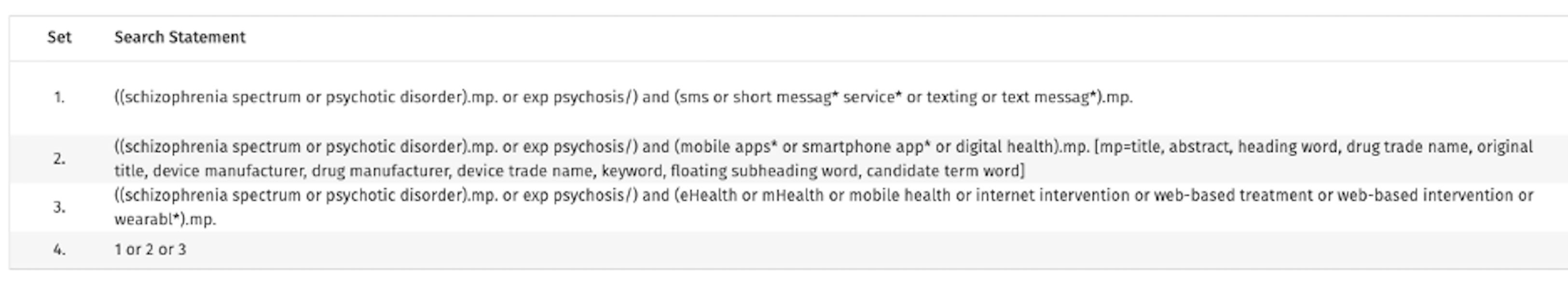

Supplement: S3 Fig — (TIFF) [file pdig.0001544.s004.tiff]
